# Assessment of Behavioral Precursors to Food Safety Practices Among Food Handlers in a Tertiary Care Hospital in New Delhi, India

**DOI:** 10.7759/cureus.86787

**Published:** 2025-06-26

**Authors:** Saurabh Chauhan, Mamta Parashar, Jyoti Khandekar

**Affiliations:** 1 Community Medicine, Vardhman Mahavir Medical College (VMMC) and Safdarjung Hospital, New Delhi, IND; 2 Community Medicine, Lady Hardinge Medical College, New Delhi, IND

**Keywords:** attitude, food handlers, food safety, knowledge, practices

## Abstract

Background: Food safety behavior modification among food handlers is pivotal to build capacity, prevent, detect, and respond to threats associated with unsafe food handling.

Objective: The objective of this study was to assess behavioral precursors to food safety practices among food handlers in a tertiary care hospital in New Delhi, India.

Materials and methods: A cross-sectional study was conducted in 2022-23 among 111 food handlers working in a tertiary care hospital in Delhi, using a pre-tested interview schedule based on the World Health Organization (WHO) food safety manual to assess behavioral precursors regarding food safety and their interrelationships. The scores obtained were graded into good, fair, and poor, and were analyzed for possible associations.

Results: The mean age of study participants was 38.64 ± 15.59 years. The majority of study participants had poor knowledge (70.3%), attitudes (88.3%), and practices (89.2%) (KAP). Training was the strongest predictor of food safety KAP, with significant positive effects across all models (β = 0.40, 0.38, 0.40; p < 0.01). Education status and work experience also showed significant associations contributing to improved food safety practices. The nature of the job significantly impacted attitudes (p = 0.04) and practices (p = 0.05), suggesting that different roles require structured interventions. The overall R² values (knowledge: 0.42, attitudes: 0.38, practices: 0.40) indicate that these predictors explain a substantial portion of the variance in KAP scores. Attitude scores were found to be significant between categories of the nature of the job (p = 0.04). Practice scores were found to be significant between various categories of educational status (p = 0.001) and categories of nature of job (p = 0.05).

Conclusion: The study assesses the possibility of evidence-based food safety interventions in educational institutions like teaching hospitals/colleges to identify knowledge gaps, address attitudinal barriers, and promote best practices through behavior modification to safeguard public health and enhance consumer confidence in the food service industry.

## Introduction

Food safety is a central concern in the food service industry, with the proficiency of food handlers in adhering to proper protocols directly impacting public health outcomes. Around the world, an estimated 600 million, almost one in 10 people, fall ill after eating contaminated food each year, resulting in 420,000 deaths and the loss of 33 million healthy life years (disability-adjusted life year (DALYs)). Food safety, nutrition, and food security are closely linked. Unsafe food creates a vicious cycle of disease and malnutrition [1,2].

The World Health Organization (WHO) estimated that in developed countries, up to 30% of the population suffers from foodborne diseases each year, whereas in developing countries, up to an estimated 70% of cases of diarrheal diseases are associated with the consumption of contaminated food [1]. The WHO aims to enhance the capacity to prevent, detect, and respond to public health threats associated with unsafe food at the global and country levels. In India, foodborne illnesses have emerged as a major health concern due to inadequate food safety provisions. In fact, out of the total outbreaks reported to the Integrated Disease Surveillance Programme (IDSP), approximately 60% are related to foodborne infection [2]. The triad of knowledge, attitude, and practices (KAP) among food handlers constitutes a focal point for scientific inquiry, as understanding these components can elucidate factors influencing food safety in various food service establishments, thus providing insights into the complexities of this critical domain.

Knowledge serves as the foundation element underpinning food safety practices among food handlers. It encompasses a broad spectrum of understanding, including microbiology, hazard identification and analysis, hygiene principles, and regulatory requirements [3]. However, the acquisition and retention of knowledge are subject to multifaceted influences, such as educational background, training efficacy, and organizational culture, necessitating rigorous scientific investigation to discern optimal knowledge dissemination strategies. Attitude constitutes a psychological construct that influences food handlers' perceptions, motivations, and behaviors toward food safety practices. Positive attitudes, characterized by a sense of responsibility and vigilance, are conducive to implementing a culture of food safety within establishments [3,4]. Practices represent the tangible manifestation of knowledge and attitudes in operational contexts, reflecting the extent to which food handlers translate theoretical understanding into actionable behaviors. Effective food safety practices encompass a spectrum of activities, such as hand hygiene, food handling procedures, temperature management, and sanitation protocols. Consistent adherence to best practices is essential for minimizing the risk of foodborne illness transmission and ensuring consumer safety [4,5].

The KAP of food handlers on food safety has received much global attention and inquiry [6-9]. However, there are limited studies that used observation to investigate the food handling practices [10-12]. Self-reported practices may not necessarily be the actual practiced food handling behavior [13], as they may include respondent bias in the study findings [14].

Food safety is a key public health priority for food service establishments, particularly for a tertiary care hospital, which majorly caters to patients, their attendants, doctors, nurses, and medical students who eat their meals outside the home [15]. Hence, food handlers can be a source of infection directly or by cross-contamination. Good hygiene and food safety practices are vital in the preparation, storage, distribution, and serving of food. Therefore, medical institutions provide a critical environment for improving food safety practices [14,15].

The current study assesses the possibility of evidence-based strategies for enhancing food safety in food service establishments, particularly in educational institutions, to identify knowledge gaps, address attitudinal barriers, and promote best practices through targeted interventions to safeguard public health and enhance consumer confidence in the food service industry, with the objective of assessing behavioral precursors among food handlers working in a tertiary care hospital in Delhi to give insights into the multifaceted determinants of food safety in food service establishments. 

## Materials and methods

Study design and settings

This cross-sectional study was conducted during 2022-2023 in New Delhi. It included all food handlers working in food establishments located within the premises of Lady Hardinge Medical College, a tertiary care teaching hospital affiliated with the University of Delhi. All 111 food handlers employed in food establishments within the campus of the tertiary care hospital were included in the study through universal sampling, based on institutional records.

Interview schedule and checklists

The data were collected using a self-designed, pretested interview schedule for assessing sociodemographic details and KAP regarding food safety (based on the WHO and Food Safety and Standards Authority of India (FSSAI) food safety manual) [1,5]. The interview schedule consisted of three sections: Section I: Sociodemographic details (age, sex, religion, education, native place, nature of job in the establishment, etc.); Section II: General information regarding functionaries of food establishments; Section III: KAP regarding food safety based on the WHO and FSSAI manual based on WHO's five keys for safer food, namely, keep clean, separate raw and cooked, cook thoroughly, keep food at appropriate temperatures, and use safe water and raw materials. The knowledge section comprised 32 questions, the attitudes section comprised 24 questions, and the practices section comprised 63 questions. The questions assessed their awareness, ranging from their personal hygiene, handwashing techniques, hygiene of the food establishments, cooking practices, food storage, packaging, and labeling KAP. Observational checklists were prepared based on standards and guidelines laid down by WHO and FSSAI for assessing the practices among food handlers in these domains.

Before the study commenced, the investigator underwent training regarding food safety from the FSSAI, Government of India. The interview schedule was pilot-tested among food handlers, and written informed consent was obtained from study participants.

The KAP of food handlers regarding food safety was based on the WHO and FSSAI food safety manuals [1,5]. A scoring system was used for the observations. Correct answers were given a score of one, and incorrect answers were given a score of 0. The scores were changed into percentages by dividing the score for each individual by the total score and multiplying it by 100. The level of KAP was then categorized into poor (<50% of total score), fair (50% - <75% of total score), and good (≥75% of total score) observations [15].

Statistical analysis

The statistical analysis of data was performed using the IBM SPSS Statistics software for Windows, version 28.0 (IBM Corp., Armonk, NY). The sociodemographic characteristics of respondents and their scores with respect to KAP were summarized using descriptive statistics. A regression model to identify predictors and one-way ANOVA were used to test the association between food safety knowledge, attitudes, and practice scores with the sociodemographic characteristics of the respondents. Findings with a p-value < 0.05 were considered to be statistically significant.

Ethical considerations

Informed consent was taken from the participants, and data confidentiality was maintained. The study was approved by the Institutional Ethics Committee (IEC) of Lady Hardinge Medical College, New Delhi, India, with letter no. LHMC/IEC/2020/19.

## Results

Sociodemographic characteristics of the study participants

The mean age of the study participants was 38.64 ± 15.59 years (range 18-60 years). The majority (88.2%) were males and practiced Hinduism (98.2%). About two-thirds (65.5%) of the participants were in the 18-28 year age group, working in the private sector. Nearly two-thirds (64.9%) of the study participants were married, while more than half of the participants had passed high school and worked as food handlers in food service establishments. Nearly half (45.9%) of the study participants had a role that involved multitasking, followed by an equal proportion of cooks and helpers (18.9%). The majority (86.5%) of total study participants have not received any training regarding food safety in the past year (Table 1).

**Table 1:** Sociodemographic characteristics of the study participants (N=111)

Characteristics	Total (N=111) [n (%)]
Age (in completed years)	
18-28	40 (36)
29-38	16 (14.4)
39-48	17 (15.4)
49-58	31 (27.9)
≥ 59	7 (6.3)
Gender	
Male	98 (88.3)
Female	13 (11.7)
Religion	
Hindu	109 (98.2)
Others	2 (1.8)
Marital status	
Married	72 (64.9)
Unmarried	33 (29.7)
Widowed	6 (5.4)
Education status	
Illiterate	5 (4.5)
Primary school	8 (7.2)
Middle school	32 (28.8)
High school	60 (54)
Graduate and above	6 (5.4)
Nature of the job	
Multitasking	51 (45.9)
Cook	21 (18.9)
Helper	21 (18.9)
Server	18 (16.3)
Training regarding food safety received in the past year	
No	96 (86.5)
Yes	15 (13.5)

**Figure 1:**
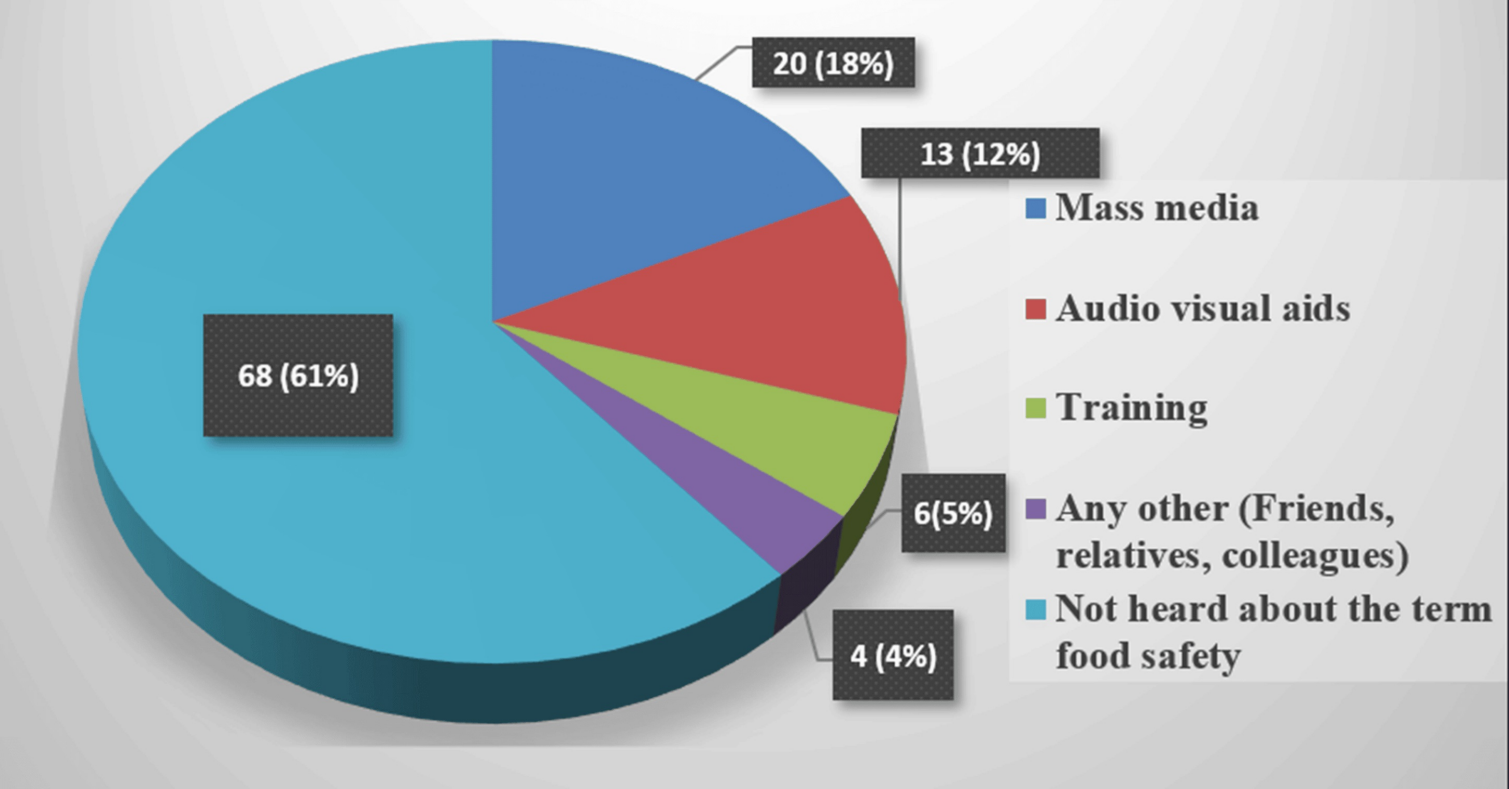
The study participants' sources of information regarding food safety (N=111)

Figure 1 describes the means of information among study participants regarding food safety. Nearly two-thirds (61%) of the respondents admitted to having never heard about food safety, while those who had heard about food safety mentioned mass media (18%) as their source of information. Table 2 describes the mean scores of the overall KAP of the study participants. The mean scores of KAP and combined KAP scores were 13.67 ± 3.31, 9.33 ± 1.77, 29.10 ± 3.97, and 52.09 ± 5.38, respectively, while the proportion of correct answers in the combined KAP, and combined KAP were 44.3, 56.3, 46.1, and 47.7, respectively, with maximum correct answers given in attitudes, which was one of the crucial factors that influenced food safety behavior, followed by practice scores, thus decreasing the occurrence of foodborne diseases, followed by knowledge and practice scores.

**Table 2:** Combined knowledge, attitudes, and practices (KAP) scores of food handlers regarding food safety (N=111)

		Proportion of correct answers	Proportion of incorrect answers
Domain	Mean ± SD (correct answers)	(%)	(%)
Combined knowledge score	42.7 ± 10.3	44.3	55.7
(Maximum obtainable score = 32)			
Combined attitude score	38.9 ± 7.4	56.3	43.7
(Maximum obtainable score = 24)			
Combined practices score	46.2 ± 6.3	46.1	53.9
(Maximum obtainable score = 63)			
Combined KAP	43.8 ± 4.5	47.7	52.3
(Maximum obtainable score = 119)			

Table 3 shows the KAP grading among study participants based on the WHO and FSSAI food safety manual, and they were graded into good, fair, and poor KAP. The majority of study participants had poor knowledge (70.3%), attitudes (88.3%), and practices (89.2%) regarding food safety, and none of them had good KAP regarding food safety.

**Table 3:** Level of knowledge, attitude and practices of the study participants regarding food safety (N=111)

Overall scores grading (%)	Knowledge*	Attitude**	Practices***
Good (>75% of total score)	0	0	0
Fair (50%-75% of total score)	33 (29.7)	13 (11.7)	12 (10.8)
Poor (<50% of total score)	78 (70.3)	98 (88.3)	99 (89.2)

Table 4 presents the regression model predicting KAP on food safety among food handlers. It examines the association between various independent variables (age, gender, education status, income, work experience, training, and nature of job) and dependent variables (KAP). The regression analysis revealed that training was the strongest predictor of food safety KAP, with significant positive effects across all models (β = 0.40, 0.38, 0.40; p < 0.01). Education status and work experience also showed significant associations, indicating that higher education levels and more experience contributed to improved food safety practices. Income had a moderate effect, while age and gender had weaker influences. Additionally, the nature of the job significantly impacted attitudes (p = 0.04) and practices (p = 0.05), suggesting that different roles require structured interventions. The overall R² values (knowledge: 0.42, attitude: 0.38, practice: 0.40) indicate that these predictors explain a substantial portion of the variance in KAP scores.

**Table 4:** Multiple linear regression model predicting knowledge, attitudes, and practices of food safety among food handlers (N=111) *p<0.01; **B: unstandardized coefficient; ***β: standardized regression coefficient

Characteristic	Knowledge			Attitudes			Practices		
	B** (95% CI)	SE	β***	B (95% CI)	SE	β	B (95% CI)	SE	β
Age (completed years)	0.10 (0.02-0.18)	0.04	0.12^*^	0.08 (0.00-0.16)	0.04	0.10	0.07 (0.01-0.13)	0.03	0.09^*^
18-28 (reference)									
29-38									
39-48									
49-58									
≥ 59									
Gender	0.15 (0.03- 0.27)	0.06	1.10^*^	0.12 (0.02-0.22)	0.05	0.08^*^	0.10 (0.02-0.18)	0.04	0.07^*^
Male (reference)									
Female									
Educational status	0.30 (0.14-0.46)	0.08	0.28^*^	0.22 (0.08-0.36)	0.07	0.20^*^	0.25 (0.11-0.39)	0.07	0.22^*^
Illiterate (reference)									
Primary school									
Middle school									
High school									
Graduate and above									
Income (in Indian rupees (INR))	0.18 (0.04-0.32)	0.07	0.15^*^	0.14 (0.02- 0.26)	0.06	0.12^*^	0.16 (0.06-0.26)	0.05	0.14^*^
Upper (7,863 and above) (reference)									
Upper middle (3,931-7862)									
Middle (2,359-3,930)									
Lower middle (1,179-2,358)									
Lower (1,179 and below)									
Work experience (years)	0.22 (0.12-0.32)	0.05	0.25^*^	0.20 (0.08-0.32)	0.06	0.22^*^	0.22 (0.12-0.32)	0.05	0.23^*^
<1 (reference)									
1-5									
>5									
Training	0.50 (0.40-0.60)	0.05	0.40^*^	0.45 (0.29-0.61)	0.08	0.38^*^	0.48 (0.32-0.64)	0.08	0.40^*^
No (reference)									
Yes									
Nature of work	0.12 (0.00-0.14)	0.06	0.11	0.05 (-0.05-0.15)	0.05	0.09	0.12 (0.02-0.22)	0.05	0.11^*^
Multitasking (reference)									
Cook									
Helper									
Server									
	R² for Knowledge Model: 0.42			R² for attitude Model: 0.38			R² for practice Model: 0.40		

Table 5 explores the factors associated with food safety knowledge, attitude, and practice mean scores among study participants. A one-way ANOVA test was applied to determine if the mean scores of KAP were different across various categories of associated factors (age, gender, education, and nature of job of study participants), and data are presented as mean ± S.D. The assumption of equality of variance failed, as shown by Levene’s statistics (p < 0.05). Knowledge scores were not found to be significantly different statistically between various categories of age, gender, and nature of the job. Attitude scores were found to be significantly different statistically between various categories of the nature of the job (Welch F (df = 3,107) = 2.74, p = 0.04). Practice scores were found to be significantly different statistically between various categories of educational status (Welch F (df = 4, 106) = 4.74, p = 0.001) and between various categories of nature of job (Welch F (df, 107) = 2.71, p = 0.05).

**Table 5:** Factors associated with mean knowledge, attitude, and practices scores of food safety among study participants (N=111) *One way ANOVA

Variable	Knowledge	Attitudes	Practices	P value*
Age (In completed years)				
18-28	13.58 ± 2.80	9.55 ± 1.70	28.78 ± 5.14	Knowledge: 0.75
29-38	14.06 ± 3.06	9.06 ± 1.91	30.38 ± 4.60	Attitudes: 0.54
39-48	14.29 ± 2.11	9.53 ± 1.71	28.82 ± 2.76	Practices: 0.71
49-58	13.32 ± 2.49	8.97 ± 1.81	28.90 ± 2.51	
>59	13.29 ± 4.07	9.86 ± 1.95	29.57± 2.14	
Gender				Knowledge: 0.69
Male	13.70 ± 2.83	9.34 ± 1.82	29.23 ± 4.17	Attitudes: 0.95
Female	13.38 ± 1.75	9.31 ± 1.31	28.08 ± 1.60	Practices: 0.32
Education status				
Illiterate	14.00 ± 1.87	9.40 ±2.40	31.40 ± 3.78	Knowledge: 0.39
Primary school certificate	14.13 ± 4.35	9.13 ± 1.35	33.50 ± 4.00	Attitudes: 0.99
Middle school certificate	12.84 ± 2.42	9.38 ± 1.71	27.94 ± 3.94	Practices: 0.001
High school certificate	13.97 ± 2.67	9.32 ± 1.88	28.72 ± 3.42	
Intermediate or post high school graduate	14.17 ± 2.71	9.50 ± 1.22	31.33 ± 5.08	
Nature of job				
Multitasking	13.20 ± 2.89	10.25 ± 3.18	28.12 ± 3.62	Knowledge: 0.19
Cook	14.57 ± 1.98	8.76 ± 1.89	30.57 ± 4.38	Attitudes: 0.04
Helper	13.43 ± 2.92	8.67 ± 1.63	29.86 ± 3.48	Practices: 0.05
Server	14.22 ± 2.60	9.50 ± 2.14	29.28 ± 4.49	

## Discussion

Most of the foodborne diseases are preventable through the proper implementation of food safety measures. Food handlers play an important role in maintaining food safety. The mean age of the study participants was found to be 38.64 ± 15.59 years (range 18-60 years). Similar mean ages of 31.7 ± 9.9 years and 45 ± 1.53 years were reported in studies conducted by Hamad et al. [16] and Bhattacharya et al. [17], respectively. A majority (88.2%) of the study participants working in food establishments were males. The findings were similar to the studies done by Acikel et al. [18] and Joseph et al. [19].

The majority of study participants were Hindus (98.2%). Prabhu et al. [20] reported similar findings where Hindus were 92%. Around two-thirds (65%) of the study participants were married. Similar findings were reported by Bou-Mitri et al. [21] and Aguomo et al. [22], where 71.7% and 61.5% of participants were married, respectively.

Nearly half (45%) of the study participants were multitaskers. This indicates that there is always a possibility that, as per the needs, the nature of the work of a person could be changed. So, while training, one has to train all food handlers regarding food safety measures as an extra precaution. This was in contrast to a study conducted by Sani et al. [23] and Lee et al. [24], where the maximum number of participants were cooks or servers, which could be due to a lack of job specifications in food establishments while recruiting employees. Also, there is no fixed nature of work at these establishments, and their nature of work keeps changing depending on the total attendance of the establishments, which keeps on fluctuating.

Less than half of the participants had good food safety knowledge and maintained good practices correctly, indicating a need for the stringent implementation of food safety guidelines. Similar studies [22-26] were conducted in which food safety KAP among food handlers was assessed.

None (0%) of the study participants had good KAP scores, which is similar to what was described by Abdul-Mutalib et al. [27] and Adesokan et al. [15], with maximum correct answers towards food safety attitudes. This could be due to the fact that attitude formation occurs through either direct experience or persuasion of others, or through mass media. As age progresses, attitude also improves through experience and direct observations, followed by practice scores, which reinforces the fact that health education intervention, if given effectively, can greatly improve the practices of the food handlers. Mean scores of the nature of the job, with attitudes and practices improved across the various categories. Similar findings were found in studies conducted by Marzban et al. [28], Rosmawati et al. [29], and Tuglo et al. [30]. 

The study highlights training as the strongest predictor of food safety KAP (p < 0.01, β ≈ 0.40), aligning with Adesokan et al. [15] and Marzban et al. [28], who emphasized the role of structured training in improving compliance. Education status and work experience also significantly influenced KAP scores, supporting findings by Bou-Mitri et al. [21] and Ncube et al. [25].

The nature of the job impacted attitudes (p = 0.04) and practices (p = 0.05), similar to Sani et al. [23] and Lee et al. [24], who noted variations in compliance based on job roles. Unlike Mutalib et al. [27], this study found no significant relationship between age and knowledge, possibly due to differences in prior training. Overall, these results emphasize the importance of continuous, role-specific training programs to enhance food safety compliance.

The strengths of the study include that it addresses a critical public health concern, as food safety in healthcare settings is paramount to prevent foodborne illnesses among vulnerable populations. The focus on a tertiary hospital in New Delhi adds relevance due to the high patient turnover and diverse population, which can provide comprehensive insights into food safety practices among food handlers. The study relies on observable practices, which adds to the reliability and eliminates social desirability bias observed in self-reported data, where participants may report what they believe is expected rather than their actual practices. The study's emphasis on behavioral precursors is significant, as understanding the attitudes and practices of food handlers can lead to more effective interventions and training programs. Moreover, the study contributes to the limited literature on food safety practices in healthcare settings in India, offering a foundation for future research and policymaking. Potential limitations of cross-sectional studies could be the generalizability of the findings, since the research is confined to a single tertiary care hospital, and the establishment of causal relationships between behavioral precursors towards food safety and bias due to some self-reported data.

## Conclusions

The assessment of KAP among food handlers in a tertiary care hospital in New Delhi, India, provides valuable insights into food safety compliance in healthcare food service settings. The findings indicate that while food handlers demonstrate a positive attitude toward food safety, significant gaps exist in both knowledge and actual practices. These gaps may contribute to unsafe food handling environments. Importantly, the study found statistically significant associations between training, education status, work experience, and improved KAP scores, suggesting that structured educational initiatives may play a critical role in enhancing food safety behavior. Given the study's cross-sectional nature, causal inferences cannot be made. However, the observed associations support that training programs have the potential, and targeted interventions dedicated to food handlers’ educational background and work roles can play a crucial role in food safety practices.

Based on the findings of this study, several recommendations can be made to improve food safety practices among food handlers in the tertiary care hospital. Firstly, regular, comprehensive training programs should be implemented to update food handlers on the latest food safety standards and practices. Secondly, it is recommended to establish a monitoring and evaluation system to ensure compliance with food safety protocols. This can include regular inspections, audits, and feedback mechanisms. Thirdly, implementing a culture of food safety within the hospital environment is crucial. This can be achieved by promoting the importance of food safety at all levels of the organization, from management to frontline staff. Additionally, it is essential to provide an adequate and regular supply of resources, such as sanitation facilities, sufficient storage equipment, and protective gear, to enable food handlers to perform their duties effectively. Implementing these recommendations can significantly enhance the overall food safety standards in the hospital, thereby safeguarding patient and consumer health.
